# Targeting abatacept-resistant T-helper-17 cells by aldehyde dehydrogenase inhibition

**DOI:** 10.1016/j.isci.2023.108646

**Published:** 2023-12-09

**Authors:** Yukiko Tokifuji, Hodaka Hayabuchi, Takashi Sasaki, Mariko Hara-Chikuma, Keiji Hirota, Hayato Takahashi, Masayuki Amagai, Akihiko Yoshimura, Shunsuke Chikuma

**Affiliations:** 1 Department of Microbiology and Immunology, Keio University School of Medicine, 35 Shinanomachi, East Lecture Hall 4F, Shinjuku, Tokyo 160-8582, Japan; 2 Center for Supercentenarian Medical Research, Keio University School of Medicine, 35 Shinanomachi, Shinjuku, Tokyo 160-8582, Japan; 3 Department of Pharmacology, Keio University School of Medicine, 35 Shinanomachi, Shinjuku, Tokyo 160-8582, Japan; 4 Laboratory of Integrative Biological Science, Institute for Life and Medical Sciences, Kyoto University, Kyoto 606-8507, Japan; 5 Department of Dermatology, Keio University School of Medicine, 35 Shinanomachi, Shinjuku, Tokyo 160-8582, Japan

**Keywords:** Molecular biology, Immunity, Cell

## Abstract

IL-17-producing helper T (Th17) cells are long-lived and serve as central effector cells in chronic autoimmune diseases. The underlying mechanisms of Th17 persistence remain unclear. We demonstrated that abatacept, a CD28 antagonist, effectively prevented the development of skin disease in a Th17-dependent experimental autoimmune dermatitis model. Abatacept selectively inhibited the emergence of IL-7R-negative effector-phenotype T cells while allowing the survival and proliferation of IL-7R^+^ memory-phenotype cells. The surviving IL-7R^+^ Th17 cells expressed genes associated with alcohol/aldehyde detoxification and showed potential to transdifferentiate into IL-7R-negative effector cells. Inhibiting aldehyde dehydrogenase reduced IL-7R^+^ Th17 cells *in vivo*, independently of CD28, and exhibited additive effects when combined with abatacept. Our findings suggest that CD28 blockade prevents inflammation without eliminating persistent memory cells. These remaining memory cells can be targeted by other drugs, such as aldehyde dehydrogenase inhibitors, to limit their survival, thereby facilitating the treatment of chronic autoimmune diseases.

## Highlights

• CD28 blockage by abatacept prevents dermatitis but does not eliminate memory Th17 cells
• Pathogenic memory Th17 cells utilize aldehyde dehydrogenases for survival
• Abatacept together with ALDH inhibitor reduce pathogenic memory Th17 cells *in vivo*

## Graphical abstract

**Figure:**
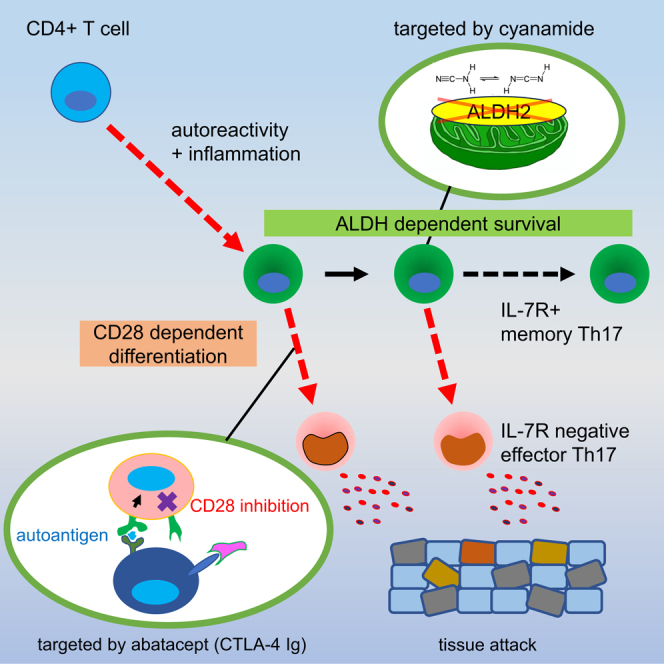

## Introduction

Autoimmune diseases affect approximately 5% of the global population.^1^ Biological drugs targeting proinflammatory cytokines are effective; however, disease recurrence during or after treatment is often problematic.^2^ One of the cellular targets of such drugs contain interleukin-17 (IL-17)-producing helper T (Th17) cells.^3^^,^^4^^,^^5^ Th17 cells regulate inflammation by producing proinflammatory effector cytokines, such as IL-17, tumor necrosis factor alpha (TNF-α), IL-22, interferon gamma (IFNγ), and granulocyte-macrophage colony-stimulating factor (GM-CSF). Furthermore, Th17 cells survive longer than other T cells. Memory-like Th17s can replicate and produce a new effector cell population, which resembles stem cells, and contributes to their persistence.^6^^,^^7^^,^^8^ Understanding the mechanisms underlying long-term survival of autoreactive Th17 will be useful for deciding the optimal treatment of chronic autoimmune diseases.

CD28, the best-known costimulatory receptor,^9^ mediates T cell activation through IL-2 production^10^ and resistance to apoptosis.^11^ At the molecular level, CD28 ligation activates serine/threonine kinase Akt, nuclear factor kB (NF-kB), and the mechanistic Target of Rapamycin (mTOR) that stimulate uptake and metabolism of glucose for full activation and differentiation into effector cells.^12^^,^^13^ Abatacept, a fusion protein of CTLA-4 and immunoglobulin (Ig) antagonizes CD28 by competing with costimulatory ligands (CD80 and CD86), acts as a strong inhibitor of T cells, and has been used for the treatment of autoimmune diseases. CD28 serves as a primary checkpoint in T cell activation but also may be involved in the peripheral maintenance of T cell homeostasis such as Th17 cells.

Desmoglein 3 (DSG3) is an adhesion molecule that is primarily expressed on keratinocytes. It is also the target autoantigen in pemphigus vulgaris, an autoimmune blistering disease.^14^ T cells extracted from mice carrying a DSG3-specific T cell receptor (Dsg3H1 TCR Tg mouse; hereafter simply designated as Dsg3H1) are known to directly infiltrate the epidermis and induce cellular immunity in DSG3-bearing keratinocytes and cause interface dermatitis after adoptive transfer into Rag2−/− mice.^15^^,^^16^

Using the modified chronic experimental autoimmune dermatitis (EAD) model, we demonstrated that CD28 signal plays a key role in activation and effector function of Th17. Abatacept treatment completely blocked the development of skin inflammation by inhibiting activation and proliferation of effector T cells. In contrast, IL-7 receptor (IL-7R)-positive Th17 cells with memory-like phenotype were resistant to abatacept and remained in the body. To inhibit abatacept-resistant remaining Th17 cells *in vivo*, we extensively characterized this population and discovered that ALDH inhibitors can prevent the formation of this memory population.

## Results

### Pathogenic Th17-dependent mouse model of chronic skin inflammation

We previously reported a Th17-dependent EAD model in mice.^16^ Briefly, naive CD4^+^ T cells extracted from Dsg3H1 mice^15^ were purified and activated *in vitro* under Th17 polarizing condition. Subsequently, when these Th17 cells were transferred into lymphocyte-deficient Rag2 knockout mice, they induced IL-17-dependent subacute skin inflammation, both histologically and immunologically resembling psoriasis.^16^ Unfortunately, severe weight loss and rapid death of recipient mice following cell transfer prevented us from analyzing persistent autoimmune disease in this model (^16^ and unpublished).

**Figure 1:**
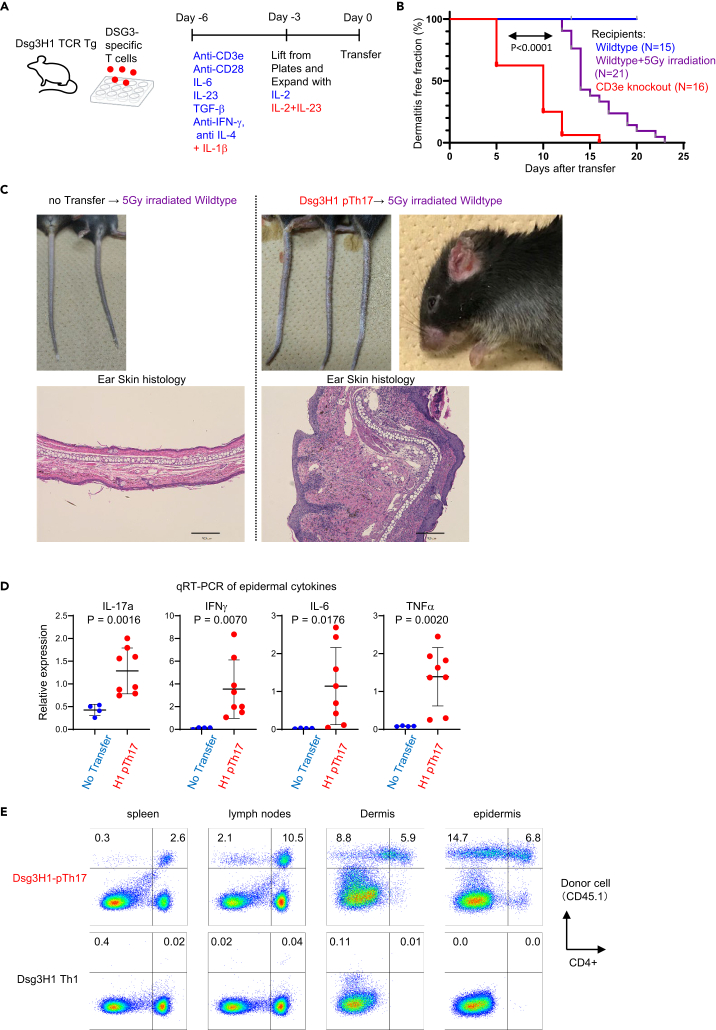
Highly polarized skin-reactive Th17 cells induce chronic dermatitis upon transfer (A) Schematic representation of the induction of pathogenic Th17 cells from desmoglein-3-specific Dsg3H1-TCR transgenic mice. Refer to the STAR methods section for details. (B) Development and kinetics of dermatitis in recipient mice. Cumulative results from three experiments. Kaplan–Meier method with log rank test. (C) Macroscopic (tail and ear) and microscopic (ear skin) views of dermatitis. Scale bars: 200 μM. (D) mRNA expression of inflammatory cytokines in epidermal tissue two weeks after transfer. Two-tailed t test (N of 4 and 7). (E) Survival of transferred T cells two weeks after transfer. Donor T cells were detected by the CD45.1 congenic marker *ex vivo*. The data represent one representative datapoint from more than 10 mice.

To investigate long-term Th17 cell survival in the persistent EAD model, we used sublethally irradiated wild-type mice as recipients. Additionally, during T cell differentiation culture, we added IL-1β, known to promote the development of long-lived pathogenic Th17 cells (pTh17; Figure 1A and STAR methods section). The resulting pTh17 cells exhibited stronger production of IL-17A compared with normally skewed Th17 cells (nTh17). Furthermore, pTh17 cells produced IL-17F (Figure S1A), which is suggested to be produced by more epigenetically committed Th17 cells.^17^ Genes encoding IL-23 receptor and GM-CSF (*Il23r* and *Csf2*, respectively) are higher in pTh17 than nTh17 (Figure S1B). Consequently, we termed the induced cells “Dsg3H1-pTh17,” representing pathogenic Th17-skewed Dsg3H1 cells.

When Dsg3H1-pTh17 cells were transferred into irradiated syngeneic wild-type C57BL/6 mice, they induced dermatitis at a slower rate than in Cd3e KO recipients (which lack endogenous T cells and showed EAD with kinetics similar to Rag2 KO recipients) (Figure 1B). The skin inflammation, typically affecting the ears, back, neck, and/or tail, persisted for at least a month without the death of recipient mice (Figure 1C and data not shown). Thickened skin with a massive infiltration of mononuclear cells in epidermal and dermal tissues was evident (Figure 1C). In the affected skin, strong expression of cytokines, such as IL-17A, IFNγ, IL-6, and TNF-α, were detected, indicating severe inflammation caused by Dsg3H1-pTh17 (Figure 1D).

Using congenically labeled donor T cells (pTh17 prepared from Dsg3H1 transgenic, CD45.1 congenic mice) allowed us to discriminate transferred cells via fluorescence-activated cell sorting (FACS) analyses in the recipients (CD45.2). In support of Th17-dependent inflammation, the transferred donor Dsg3H1-pTh17 cells (CD45.1^+^) were detected in epidermal and dermal tissues after 2 weeks, as well as in skin-draining lymph nodes and spleen (Figure 1E, upper panels). In contrast, Dsg3H1 T cells skewed into Th1 (Dsg3H1 Th1; Figure 1E lower panels) did not persist in the skin nor induce skin inflammation *in vivo*. Moreover, Dsg3H1-pTh17 cells neither caused any inflammation nor exhibited survival beyond 2 weeks *in vivo* (data not shown) in non-irradiated wild-type mice. Taken together, we have successfully developed a chronic EAD model induced by the injection of pathogenic Th17 cells reactive to a defined autoantigen in the skin.

### CD28 blockade by abatacept prevents Th17-mediated skin inflammation

**Figure 2:**
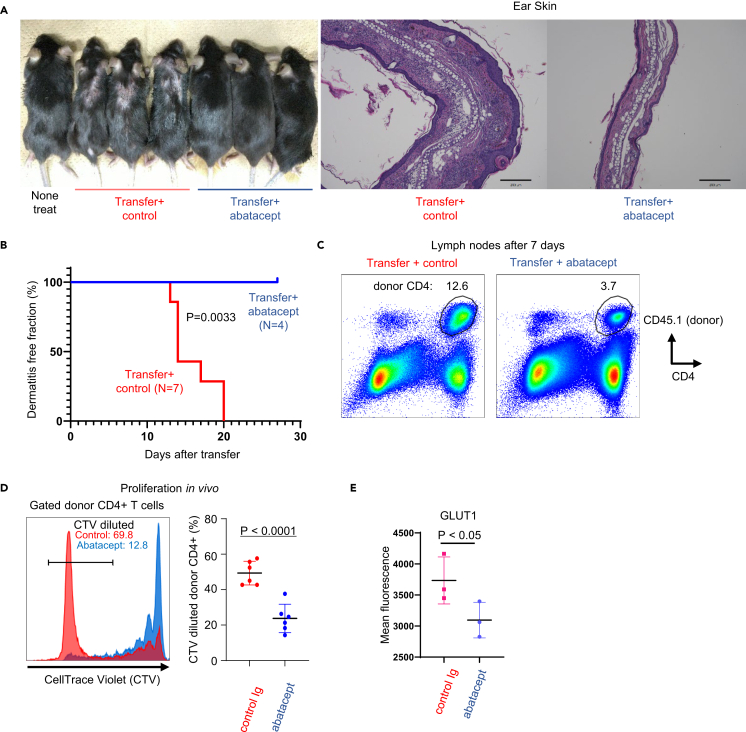
Prevention of dermatitis by abatacept, a CD28 antagonist (A and B) Development and kinetics of dermatitis. A group of mice received human CTLA-4 Ig (abatacept; 200 μg/body, every 3 days) after the transfer of CellTrace Violet (CTV)-labeled pTh17 cells prepared from Dsg3H1-TCR Tg CD45.1 congenic mice. Control mice received the same dose of human Ig. Data from three independent experiments. (C and D) *In vivo* detection of donor cell proliferation one week after transfer. Gated CD4^+^ CD45.1^+^ donor cells (C) were evaluated for CTV dilution (D, left histogram), and the proportion of CTV-diluted cells is shown in (D). (N = 6 each) (E). Gated donor cells (C) were stained with Glut1 antibody (N = 3 each). Two-tailed t test.

Abatacept, a human CTLA-4 Ig that inhibits CD28 signaling, was previously shown to ameliorate human psoriasis.^18^^,^^19^ However, its impact on skin-reactive helper T cells remains unknown. Therefore, we conducted tests using abatacept in our model. Remarkably, mice that received Dsg3H1-pTh17 cells and were treated with abatacept showed nearly complete prevention of skin lesions, indicating the critical role of CD28 signaling in skin inflammation (Figures 2A and 2B).

To facilitate tracking of transferred cells and assessment of proliferative responses, we utilized congenic marker (CD45.1) and a proliferation reporter dye (CTV) (Figures 2C and 2D). Abatacept-treated recipients showed fewer donor cells compared with control mice (Figure 2C). While a significant increase in the proliferation of donor CD4^+^ cells was observed in mice treated with control Ig, abatacept-treated mice exhibited inhibition of proliferative responses in transferred pTh17 cells (Figure 2D). Glucose transporter 1 (GLUT1), a target gene of CD28 and a hallmark indicator of glucose metabolism and extensive T cell proliferation,^12^^,^^13^ showed significantly lower expression in donor cells derived from abatacept-treated recipients (Figure 2E). These findings suggest that abatacept exerts an inhibitory effect on pTh17 cells.

### CD28 drives expression of proinflammatory genes in pathogenic Th17

**Figure 3:**
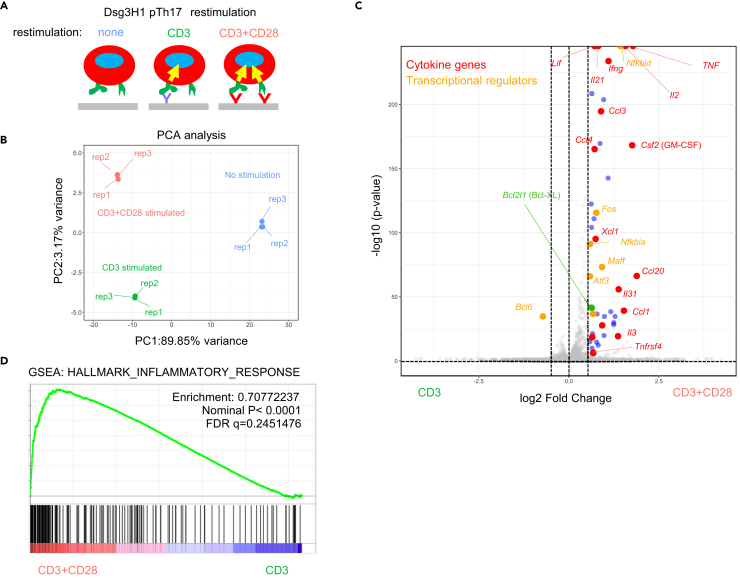
CD28 drives the proinflammatory effector function of pTh17 cells (A) Scheme of a short-term (2 h) restimulation of pTh17 cells. (B) Principal-component analysis of RNA-sequencing data after restimulation (N = 3 each). (C) Volcano plot comparing RNA-sequencing data of CD3- vs. CD3^+^CD28-stimulated cells. Notably, the presence of CD28 signal greatly augmented the expression of cytokine genes (), and transcriptional regulators involved in the inflammatory response ().  represent significant differentially expressed genes (DEGs) (log2Fc > 1.5, *P*_adj_ < 0.05). (D) Gene set enrichment analysis of log-normalized data from CD3- vs. CD3^+^CD28-stimulated cells. This RNA-sequencing experiment was performed once.

The robust effects of abatacept prompted further investigation into the fundamental roles of CD28 signaling in pTh17 cells. Therefore, we performed an RNA-sequencing analysis on fully differentiated Dsg3H1-pTh17 cells that were restimulated with plate-immobilized antibodies (Figure 3A). Importantly, the “CD3-stimulated” and “CD3+CD28-stimulated” samples showed distinct clustering in principal-component analysis of RNA-sequencing data, as early as 2 h after restimulation (Figure 3B).

Analysis of differentially expressed genes (DEGs) revealed that the rapid induction (at 2 h) of effector cytokines and transcriptional factors critically depend on CD28 signaling (Figure 3C). Specifically, we found that most CD28-dependent genes were cytokines (including those encoding IL-21, IL-2, TNF-α, GM-CSF, CCL4, XCL1, CCL20, IL-2, IL-31, and Tnfrsf4) and transcriptional regulators (*Nfkbid*, *Fos*, *Nfkbia*, *Maff*, and *Atf3*) that play pivotal roles in inflammation. These genes coexisted with an antiapoptotic protein, Bcl-XL (encoded by *Bcl2l1*) (Figure 3C).

A gene set enrichment analysis (GSEA) demonstrated that “CD3+CD28-stimulated cells” exhibited a strong bias toward the “INFLAMMATORY RESPONSE” signature compared with cells stimulated by CD3 alone (Figure 3D). We confirmed these findings by reactivating Dsg3H1-pTh17 cells using the physiologic cognate peptide recognized by Dsg3H1 T cells^15^ (Figure S2A). We observed that secondary proliferation (Figure S2B) and the production of effector cytokines (Figure S2C) were significantly augmented by CD28 signaling. These findings indicate that signals mediated by CD28 are critically involved in the secondary response of pTh17 cells.

### Abatacept inhibits IL-7R^neg^ inflammatory T cells but not IL-7R^pos^ memory T cells

**Figure 4:**
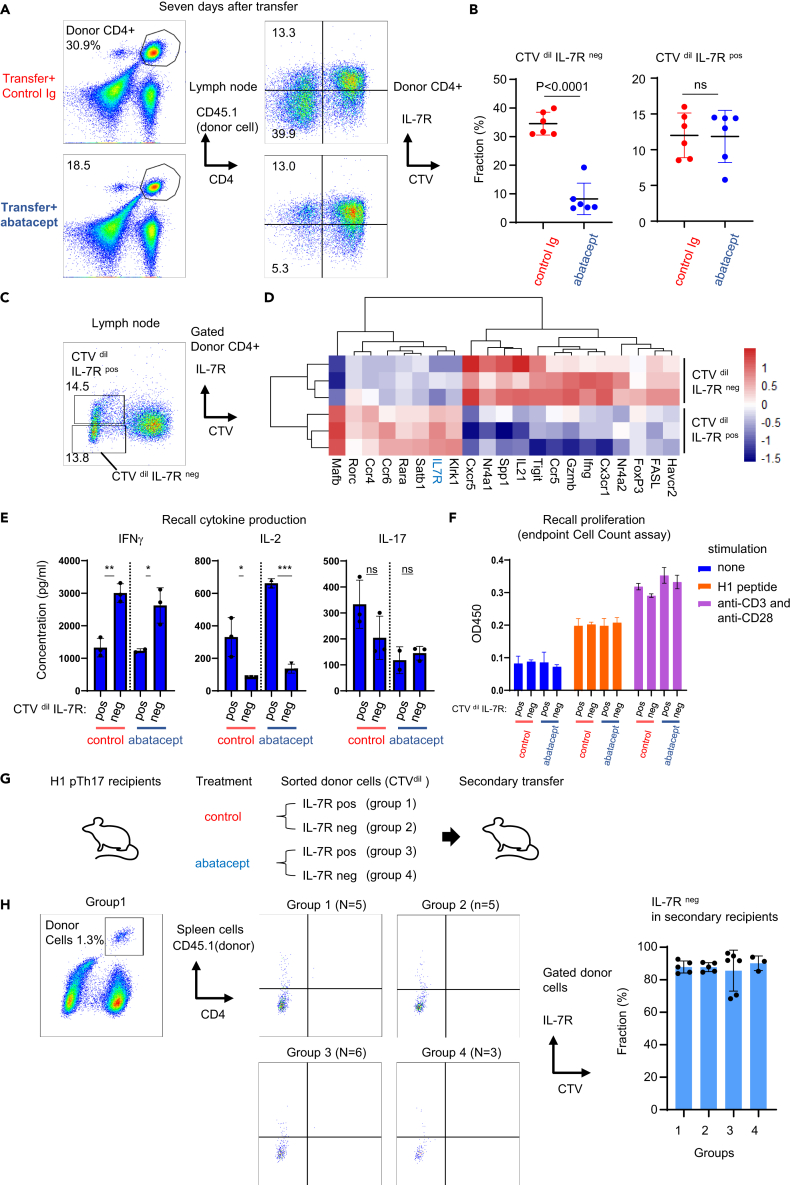
CD28 blockade inhibits IL-7R^neg^ inflammatory T cells but not IL-7R^pos^ memory-phenotype Th17 cells (A and B) Transferred donor T cells were examined for the expression of IL-7R. Abatacept significantly prevented the emergence of CTV-diluted (dil) IL-7R-negative (neg) population (B, left) but showed no effects on the IL-7R-positive (pos) counterpart. (Two-tailed t test, N of 6 and 7, three experiments). (C) Cell sorting for RNA sequence. (D) A heatmap showing cytokines, chemokines, receptors, and transcriptional factors differentially expressed between CTV^dil^ IL-7R^neg^ and CTV^dil^ IL-7R^pos^ donor cell populations. (N = 3 each, one experiment). (E and F) Cytokine production and proliferation of donor T cells recovered from treated recipient mice. (G and H) A scheme (G) and the result (H) for a serial transfer experiment. Two experiments.

Our model allowed us to examine the phenotypes of transferred pTh17 cells following CD28 blockade by abatacept. One week after transfer, we conducted an extensive analysis of donor Dsg3H1-pTh17 cells isolated from lymph nodes using a combination of congenic markers (CD45.1), a proliferation reporter dye (CTV), and other markers associated with effector/memory responses. We observed that the majority of proliferated CTV-diluted (CTV^dil^) cells exhibited a low/negative (^neg^) phenotype for the IL-7 receptor α (hereafter referred to as IL-7R), and those from abatacept-treated mice were much fewer in number compared with control mice (Figures 4A and 44B left panels). In contrast, donor-derived cells that survived in abatacept-treated mice were mostly IL-7R positive (^pos^). Moreover, the number of CTV^dil^ IL-7R^pos^ cells was not affected by abatacept, indicating that these cells proliferated independently of CD28 signaling (Figure 4B right).

We compared transcriptome data between the CTV^dil^ IL-7R^pos^ and CTV^dil^ IL-7R^neg^ populations in donor cells isolated from control mice (Figure 4C). Genes highly expressed by CTV^dil^ IL-7R^neg^ cells included effector molecules (such as *Gzmb* and *Fasl*), cytokines (*Il21*, *Ifng*, *Spp1*; osteopontin), chemokine receptors (*Cxcr5* and *Cx3cr1*), transcription factors (TF) (*Nr4a1* and *Nr4a2*), and cell surface molecules (*Havcr2* and *Tigit*), all of which suggested strong T cell activation (Figure 4D). In contrast, IL-7R^pos^ cells expressed genes related to Th17 cells (*Satb1*, *Mafb*, *Ccr4*, *Ccr6*, and *Rara*; Figure 4D). These findings suggested that inflammatory cells in the IL-7R^neg^ population were inhibited by abatacept. To directly examine cytokine expression, we sorted CTV^dil^ IL-7R^pos^ and CTV^dil^ IL-7R^neg^ cells from recipients treated with control Ig or abatacept. An equal number of sorted cells were then restimulated with the cognate antigenic Dsg3H1 peptide. Regardless of the treatment, CTV^dil^ IL-7R^neg^ cells produced higher levels of IFNγ, whereas CTV^dil^ IL-7R^pos^ cells expressed IL-2 (Figures 4E and 4F). Both populations produced a comparable amount of IL-17A, indicating that they are subpopulations of Th17 cells (Figure 4E). Importantly, the sorted cells proliferated similarly (Figure 4F), indicating that unlike naive T cells, Th17 cells did not become anergic following CD28 blockade.

We conducted experiments to investigate whether IL-7R-positive and -negative pTh17 cells can transdifferentiate into each other and set up a transfer experiment into secondary recipients (Figure 4G). We observed that most CTV^dil^ IL-7R^pos^ cells, when transferred into secondary recipients, became IL-7R^neg^, whereas most CTV^dil^ IL-7R^neg^ cells remained IL-7R^neg^ (Figure 4H). Taken together, these results suggested that pTh17 cells comprise two distinct populations. Abatacept appeared to block the IL-7R^neg^ effector-like Th17 cells but allowed the survival of IL-7R^pos^ memory-like cells that have the potential to transdifferentiate into IL-7R^neg^ effector cells.

### Abatacept inhibits effector signature but allows survival of persistent memory cells

**Figure 5:**
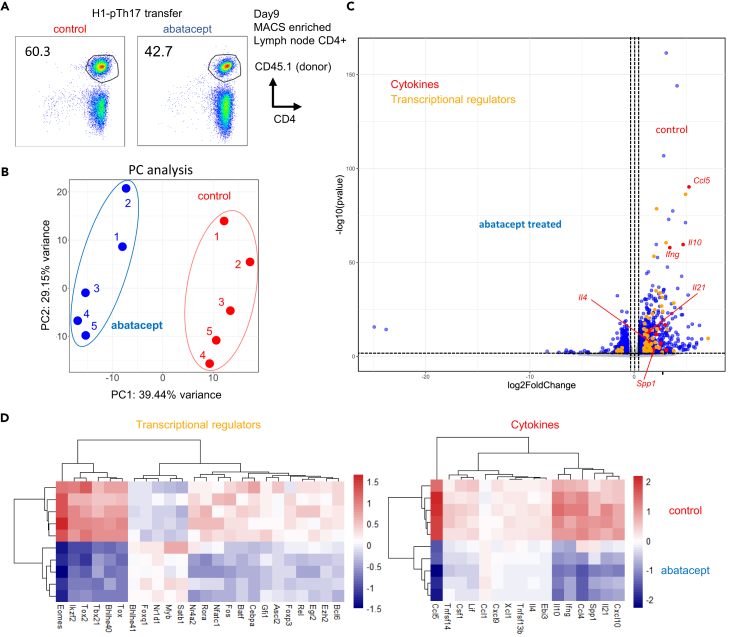
CD28 blockade inhibits effector function but leaves unique memory-phenotype T cells (A) Sorting strategy for RNA-sequencing analysis. CD4^+^ T cells from recipients were first enriched magnetically, and CD4^+^ CD45.1^+^ donor T cells were purified by FACS. (B) Principal-component analysis of RNA-sequencing data. (N = 5 each). (C) A volcano plot of RNA-sequencing data representing genes expressed in CD4^+^ donor T cells. Cytokine genes () and transcriptional regulators () are highlighted.  represents significant DEGs (log2Fc > 2, *P*_adj_ < 0.05). (D) Heatmaps demonstrating the expression of cytokine genes and transcriptional regulators. The experiment was performed once.

We conducted RNA sequencing, to directly compare the entire donor T cell population isolated from recipients treated with either control Ig or abatacept (Figures 5A and 5B). As anticipated from the *in vitro* data, cells from abatacept-treated mice showed decreased expression of cytokines and transcription factors (TFs) associated with effector function (Figures 5C and 5D). The cytokines downregulated by abatacept treatment included those typically expressed by activated helper T cells, such as *Ifng* (Th1), *Il4* (Th2), *Il21* (T follicular helper; Tfh), and *Spp1*. The TFs downregulated by abatacept also encompassed lineage-specific TFs such as *Tbx21* (T-bet; Th1), *Foxp3* (Treg), *Bcl6* (Tfh), and those involved in activation and function (*Eomes*, *Tox*, *Tox2*, *Nfatc1*, *Nr4a2*, *Ezh2*, and *Batf*), indicating a clear inhibition of effector function.

Conversely, T cells recovered from abatacept-treated recipients did not show significant upregulation of cytokines or chemokines, except for *Ccl1*. However, they exhibited upregulation of unique transcriptional factors (*Nr1d1*, *Satb1*, *Bhlhe41*, *Myb*, and *Foxq1*). SATB1^20^ and NR1D1 (REV-ERBα)^21^^,^^22^^,^^23^ have been reported to be involved in the development and function of Th17 cells. Myb is known to be essential in CD62L^pos^ stem cell memory development.^24^^,^^25^ These findings collectively support the notion that CD28 blockade by abatacept inhibited the effector function of Th17 cells while preserving a unique IL-7R^pos^ memory population.

### Abatacept-resistant memory Th17 cells exhibit genes for aldehyde dehydrogenases

**Figure 6:**
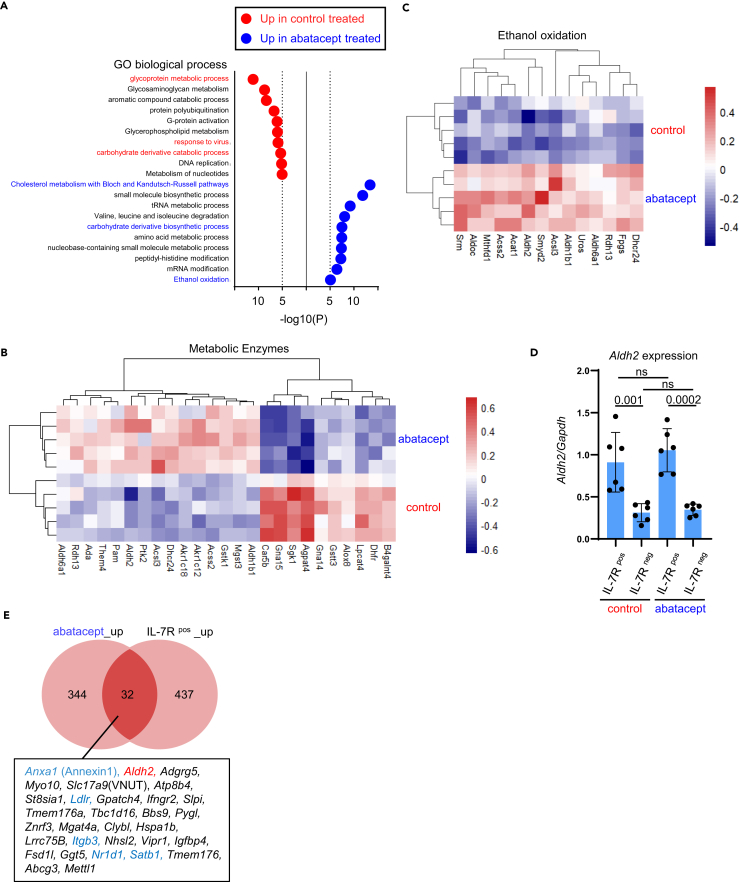
CD28-independent memory Th17 cells express aldehyde dehydrogenases (A) Gene ontology analysis of RNA-seq data (Figure 5). A graph of enriched terms across lists of DEGs for “Enzyme” categorized by Ingenuity Pathway Analysis, upregulated in control () and abatacept treatment (). (B) Heatmap showing DEGs categorized as “cytosolic enzymes.” (C) Heatmap showing DEGs categorized as “ethanol oxidation”. (D) Quantitative reverse transcription PCR of *Aldh2* gene from control and abatacept-treated mice (N = 6.) One-way ANOVA (p values are indicated in the graph. ns: not significant). (E) Venn diagram comparing DEGs upregulated in “abatacept” and “IL-7R ^pos^” group.

We further investigated the genes that may function on T cells extracted from abatacept-treated mice. Cells from control mice showed genes associated with “glycoprotein metabolic process,” “response to virus,” and “carbohydrate derivative catabolic process” signatures, suggesting the reliance of the cells depends on glycolysis for proliferation and effector function (Figure 6A). In contrast, cells from abatacept-treated mice showed genes linked to “cholesterol metabolism pathways,” “carbohydrate biosynthesis process,” and “amino acid metabolism process,” including *Dhcr24*, *Acsl3*, *Them4*, and *Acss2* (Figures 6A and 6B). These findings align with previous reports that highlight the importance of cholesterol and lipid metabolism in the survival and pathogenicity of Th17 cells.^26^^,^^27^

In addition to genes involved in energy acquisition, we observed a significant upregulation of genes related to alcohol metabolism in donor cells treated with abatacept (categorized in “ethanol oxidation”; *Aldh2*, *Acss2*, *Aldh1b1*, *Acat1*, *Fpgs*, *Uros*, *Mthfd1*, *Acsl3*, *Aldoc*, *Aldh6a1*, etc.; Figure 6C). We were particularly intrigued by the upregulation of aldehyde dehydrogenase (ALDH) genes (*Aldh2*, *Aldh1b1*, and *Aldh6a1*) for several reasons. First, ALDH plays a role in regulating stemness in hematopoiesis^28^ and malignancy.^29^ Second, ALDH is involved in mitochondrial function.^30^ Third, ALDH may contribute to T cell survival.^31^ Fourth, ALDH expression has been reported in supporting Treg survival in humans.^32^ Importantly, we also found that *Aldh2* was upregulated in IL-7R^pos^ donor T cells extracted from both control and abatacept-treated mice (compared with IL-7R^neg^ donor T cells.) (Figure 6D) Consequently, *Aldh2* was upregulated in both IL-7R^pos^ cells and T cells isolated from abatacept-treated mice (that are enriched in IL-7R^pos^ memory cells in independent cohorts (Figure 6E). Therefore, our model reveals that memory-phenotype pTh17 cells exhibit a unique metabolic pathway that may involve ALDH for both survival and function.

### Abatacept together with ALDH inhibitor targets memory Th17 cells

**Figure 7:**
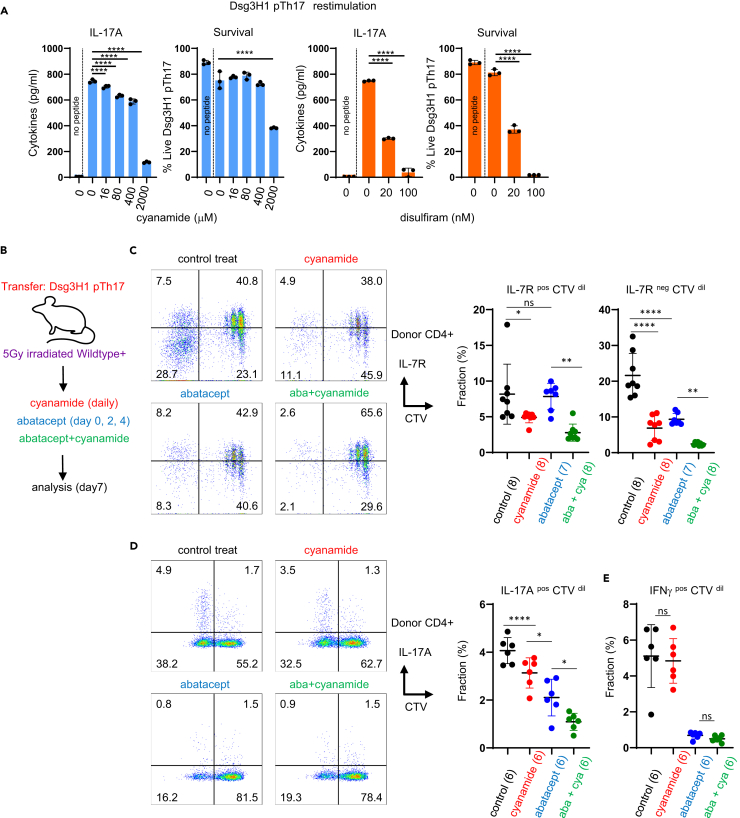
Cyanamide, an ALDH inhibitor, inhibits memory-like Th17 cells independently of CD28 blockade (A) Effects of an ALDH inhibitor on pTh17 cells *in vitro*. Dsg3-H1 pTh17 cells were restimulated *in vitro* with or without inhibitors. Three days later, cytokine production and cell viability were evaluated. Multiple comparisons by one-way ANOVA using no inhibitor as control (∗∗∗∗; p < 0.0001). (B) Treatment of EAD mice with an ALDH inhibitor *in vivo*. Groups of mice were treated with daily cyanamide (cya) with or without abatacept (aba). (C and D) FACS analysis. For (D), lymph node cells from the treated mice were isolated and stimulated *ex vivo* to induce cytokines (see STAR methods). IL-17A^pos^ (C) and IFNγ^pos^ (E) cells were analyzed using a separate staining. One-way ANOVA (∗p < 0.05, ∗∗p < 0.001, ∗∗∗∗p < 0.0001). The number of mice is indicated in the graphs. Each experiment was performed two times.

We aimed to explore the role of ALDH in Th17 activity both *in vitro* and *in vivo*. We used cyanamide and disulfiram, which are traditionally used as anti-alcoholic drugs. Treating DsgH1-Th17 cells with cyanamide or disulfiram inhibited cytokine production at lower doses and induced cell death at higher doses (Figure 7A). Subsequently, we treated EAD mice with cyanamide, either alone or in combination with abatacept (Figure 7A). We observed that mice treated with cyanamide showed a marked reduction in both IL-7R^pos^ CTV^dil^ and IL-7R^neg^ CTV^dil^ donor cells (Figure 7B). In contrast, abatacept alone selectively reduced the IL-7R^neg^ CTV^dil^ population (but not IL-7R^pos^ CTV^dil^) (Figure 7C). Notably, recipient mice treated with a combination of abatacept and cyanamide showed fewer CTV^dil^ cells than those receiving single treatments, suggesting an additive effect.

Finally, we confirmed the effects of the two drugs by examining actual cytokine expression in CTV^dil^ cells. As presented in Figure 7D, mice treated with abatacept or cyanamide alone showed a significant reduction in IL-17-producing cells, and those treated with the combination showed an additive effect. In contrast to IL-17, IFNγ production was almost completely inhibited by abatacept alone, whereas cyanamide alone or in combination with abatacept had minimal effects on IFNγ (Figure 7E).

These findings suggested that the inhibition of ALDH and CD28 affects self-reactive pathogenic Th17 cells through distinct mechanisms. ALDH inhibition had inhibitory effects on the survival and IL-17 expression of IL-7R^pos^ memory Th17 cells, whereas CD28 inhibition primarily affected the differentiation into effector Th17 cells. Importantly, the combination of both treatments had the most pronounced effect in reducing both memory and effector Th17 cell populations.

### ALDH2 correlates with IL-17 production in human cancer

**Figure 8:**
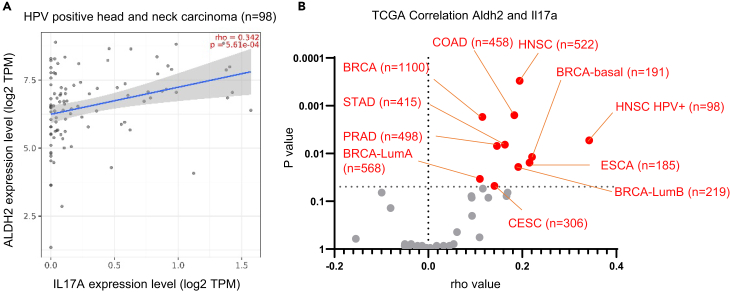
ALDH2 associates with Th17 in cancer (A) TCGA database analysis by Timer 2.0 platform reveals a significant correlation between *ALDH2* and *IL-17A* expression (HPV-positive head and neck carcinoma as an example). (B) Comprehensive analysis of *ALDH2* and *IL-17A* expression in various types of cancers. For the analysis of gene correlation, partial Spearman correlation was determined through TIMER 2.0 analysis.^45, 46^ Note that all cancer types that showed p < 0.05 represented a weak but positive correlation between *ALDH2* and *IL-17A* expression, indicative of potential infiltration. BRCA, breast invasive carcinoma; CESC, cervical squamous cell carcinoma and endocervical adenocarcinoma; COAD, colon adenocarcinoma; ESCA, esophageal carcinoma; HNSC, head and neck squamous cell carcinoma; LumA, luminal A; LumB, luminal B; PRAD, prostate adenocarcinoma; STAD, stomach adenocarcinoma.

Lastly, we investigated whether ALDH2 expression is functionally correlated with Th17 activity in humans, particularly in a cancer context. Upon re-examining of The Cancer Genome Atlas data, we observed a weak but significant correlation between *ALDH2* and the *IL-17A* gene in certain types of cancers (Figure 8). Notably, *IL-17A* expression was evident in a limited fraction of patients, and it showed a significant correlation with high *ALDH2* expression. Although these findings are preliminary, they suggest that ALDH2 may play a role in Th17 activity in humans.

## Discussion

We demonstrated that CD28 blockade selectively inhibits effector Th17 cells that are highly differentiated, leading to the complete inhibition of dermatitis. Traditionally, it has been thought that naive T cells that receive TCR signals without CD28 activation become anergic or unresponsive, thereby contributing to tolerance.^9^ Given that the CD28/PI-3K/AKT axis is a hallmark of glycolysis,^12^ it is plausible that abatacept inhibits aerobic glycolysis, which is essential for extensive proliferation and the expression of effector cytokines. However, we observed that abatacept did not inhibit the proliferation of memory-like T cells. This abatacept-resistant memory-phenotype Th17 population may explain the persistence of the disease, leading to recurrence during or after treatment. Recently, certain immunosuppressants, such as rapamycin,^33^ MEK inhibitors,^34^ and tyrosine kinase inhibitors,^35^ have been found to induce long-term memory populations and sustain chronic immune responses. Therefore, in the context of autoimmunity, a potential drawback of CTLA-4 Ig is that although it may ameliorate inflammation by blocking CD28, it could also generate persistent, long-term memory Th17 cells by preventing exhaustion. Indeed, our data, along with previous research, indicate that memory-like Th17 cells can give rise to pathogenic effector cells (as shown in our data and by others^6^).

Regarding the mechanisms underlying the persistence of Th17 cells, Muranski et al.^8^ have suggested the possibility of stemness, whereas Karmaus et al.^7^ have proposed the existence of two metabolically distinct populations. Our data support the existence of two distinct Th17 populations that show different responses to CD28 blockade.

Interestingly, in our study, the remaining memory-like Th17 cells expressed ALDH genes and can be targeted through systemic inhibition of ALDH. Therefore, ALDH not only controls stem cells but is also involved in the detoxification of endogenously produced aldehydes. Notably, the failure to detoxify endogenously produced aldehydes in patients with a combination of the ADH5 allele and ALDH2 causes Fanconi anemia, underscoring the critical role of ALDH in the hematopoietic system.^36^^,^^37^^,^^38^ Aldehydes are known to inhibit T cells, as exemplified by excessive alcohol consumption negatively impacting follicular helper T cells and attenuating immune responses.^39^^,^^40^ *Aldh2*-deficient mice show impaired T cell responses, which are associated with altered metabolism.^31^ Our data suggested that the inhibition of ALDH may lead to increased intracellular aldehyde concentrations in memory T cells, potentially affecting immune function.

An important question arises: does the genetic diversity of ALDH genes influence T cell immune responses in humans? The most well-known single-nucleotide polymorphism (SNP) in the ALDH2 gene causes loss of function and is predominantly found in the East Asian population.^41^ Despite the population with this SNP having low ethanol consumption, it is associated with cancer susceptibility and progression. This suggests that the detoxification of endogenous aldehydes by ALDH is not negligible in tumorigenesis and progression. Furthermore, our preliminary data demonstrated a positive correlation between *ALDH2* and *IL-17A* expression in certain cancers, such as head and neck carcinoma, colon adenocarcinoma, and esophageal cancer. This finding suggested a potential contribution of ALDH activity to memory or effector Th17 responses, which may be beneficial in the context of cancer.

In conclusion, we demonstrated a unique role of ALDH regulating Th17 cell responses. This systemic control of ALDH may hold promise for designing future treatment strategies for diseases involving T cell responses.

### Limitations of the study

Firstly, we used irradiated wild-type mice as recipients rather than Rag2 knockout mice. Consequently, we did not address the potential contributions of recipient-derived lymphocytes in the establishment of EAD, including epitope spreading, autoantibody production, and T regulatory cell activity.

Secondly, although we demonstrated the persistence of CTV^dil^ IL-7R^pos^ cells after abatacept treatment and their ability to produce IL-7R^neg^ effector cells, the limited cell numbers prevented us from directly confirming whether this phenomenon contributed to disease recurrence after discontinuing treatment.

Thirdly, the effects of abatacept and ALDH inhibitors on tissue resident memory T cells remain unclear and warrants further investigation. Fourthly, in terms of clinical relevance, comprehensive analyses examining whether ALDH expression is functionally correlated with Th17 cells in human autoimmune dermatitis or other autoimmune diseases are currently lacking. Future studies should address these points to provide a more comprehensive understanding of the topic.

## STAR★Methods

### Key resources table

**Table:** 

REAGENT or RESOURCE	SOURCE	IDENTIFIER
**Antibodies**		

Functional grade anti-mouse CD3e	Thermo Fisher	Cat#16-0031-82; RRID:AB_468847
Functional grade anti-mouse CD28	Thermo Fisher	Cat#16-0281-82; RRID:AB_468921
Functional grade anti-mouse IL-4	Thermo Fisher	Cat#16-7041-85; RRID:AB_469208
Functional grade anti-mouse IFNγ	Thermo Fisher	Cat# 16-7311-81; RRID:AB_469242
FITC anti-mouse CD4	Thermo Fisher	Cat#11-0042-82 ; RRID:AB_464896
APC anti-mouse IL-17A	Thermo Fisher	Cat#17-7177-81 ;RRID: AB_763580
PE anti-mouse IFNγ	Thermo Fisher	Cat#12-7311-82 ;RRID: AB_466193
PE anti-mouse IL-17F	BioLegend	Cat#517007 ;RRID: AB_ 10661730
APC anti-mouse CD45.1	Thermo Fisher	Cat#17-0453-82 ;RRID: AB_469398
PerCP-Cy5.5 anti-mouse CD4	Thermo Fisher	Cat#45-0042-82 ;RRID: AB_1107001
PE anti-mouse CD127	Thermo Fisher	Cat#12-1271-82 ;RRID: AB_465844
PE anti-mouse IL17A	Thermo Fisher	Cat#12-7177-81 ;RRID: AB_763582
Alexa Fluor 647 anti GLUT1 antibody	Abcam	Cat#ab195020

**Chemicals, peptides, and recombinant proteins**		

Recombinant mouse IL-1β	Peprotech	Cat#211-11b
Recombinant mouse IL-6	Peprotech	Cat#216-16
Recombinant mouse IL-2	Peprotech	Cat#212-12
Recombinant mouse IL-23	BioLegend	Cat#589002; RRID: AB_10663413
Recombinant human TGF-β1	Peprotech	Cat#100-21
Recombinant mouse IL-12	Peprotech	Cat#210-12
abatacept	Bristol Myers Squibb	N/A
Human Immunoglobulin	Jackson ImmunoResearch Laboratories	Cat#009-000-003
CD4(L3T4) MicroBeads mouse	Miltenyi Biotec	Cat#130-117-043
DSG-H1 mimotope (RNKAEFHQSVISQYR)	Synpeptide(Sanghai)	
cyanamide	Fujifilm-Wako Pure Chemical Corp.	Cat#030-15231
disulfiram	Tokyo Chemical Industry	Cat#B0479
CellTrace Violet	Thermo Fisher	Cat#C34571
Cell Count Reagent SF	Nacalai Tesque Inc.	Cat# 07553-15
Hyaluronidase	Fujifilm-Wako Pure Chemical Corp.	Cat#087-10481
Trypsin/1 mmol/L-EDTA Solution	Nacalai Tesque	Cat#32777-44
Collagenase D	Roche	Cat#11088858001
DNAse-I	Roche	Cat#11284932001
Brefeldin A Solution (1000X)	Thermo Fisher	Cat#00-4506-51
Monensin Solution (1000X)	Thermo Fisher	Cat#00-4506-51
Fixable Viability Dye eFluor™ 780 (FVD)	Thermo Fisher	Cat#65-0865-14

**Critical commercial assays**		

RNeasy plus micro Kit	Qiagen	Cat#74034
BD™ Cytometric Bead Array (CBA) Mouse Th1/Th2/Th17 CBA Kit	BD Bioscience	Cat#560485; RRID: AB_2869354
High-Capacity cDNA Reverse Transcription Kit	Thermo Fisher	Cat#4368814
SsoFast EveGreen Supermix	Bio-Rad	Cat#1725201

**Deposited data**		

RNA sequence	DDBJ	#DRA016062 (run number DRR457517-DRR457541)

**Experimental models: Organisms/strains**		

B6 CD3e knockout	Sommers et al.^42^	N/A
DsgH1 TCR-Tg	Takahashi et al.^15^	N/A
B6 CD45.1 congenic	RIKEN Bioresource Center, JAPAN	Strain #:RBRC00126
C57BL/6NCrSlc	Sankyo Laboratories	N/A

**Oligonucleotides**		

Primer for quantitative reverse transcription (RT)-PCR: Il23r forward: caggaggaaaccagcatcgt	This paper	N/A
Primer for RT-PCR: Il23r reverse: tcccaattgccaaacaggaga	This paper	N/A
Primer for RT-PCR: Csf2 forward: cagggtctacggggcaattt	This paper	N/A
Primer for RT-PCR: Csf2 reverse: gtgtttcacagtccgtttccg	This paper	N/A
Primer for RT-PCR: Aldh2 forward: agaccatcgaggaggttgtg	This paper	N/A
Primer for RT-PCR: Aldh2 reverse: ctgccactccctgacatctt	This paper	N/A
Primer for RT-PCR: Gapdh forward: atgaatacggctacagcaacagg	This paper	N/A
Primer: Gapdh reverse: ctcttgctcagtgtccttgctg	This paper	N/A

**Software and algorithms**		

GraphPad PRISM software (ver. 8.4.3)	GraphPad software	N/A

**Other**		

LS columns	Miltenyi Biotec	Cat#130-042-401

### Resource availability

#### Lead contact

Further information and requests for resources and reagents should be directed to and will be fulfilled by the Lead Contact, Shunsuke Chikuma (schikuma@keio.jp).

#### Materials availability

All unique reagents used in this study are available from the lead contact upon reasonable request.

#### Data and code availability

(1) RNA-seq data have been deposited at DDBJ and are publicly available as of the date of publication.
(2) Accession numbers are listed in the key resources table. This paper does not report original code.
(3) Any other information required to reanalyze the data reported in this paper is available from the lead contact upon request.

### Experimental model and study participant details

#### Animals

Dsg3H1 TCR Tg,^15^ B6 CD45.1 congenic mice (Jackson Laboratory), and CD3 epsilon knockout mice were used in this study. Female mice, 7 weeks old, of the wild-type C57BL/6NCrSlc strain were obtained from Sankyo Laboratory (Tokyo, Japan). All mice were housed in SPF facilities at Keio University under standard conditions, which included a12-h light–dark cycle (lights-on at 7:30 a.m.) at a temperature of 24°C ± 1°C, with food and water provided *ad libitum*. All animal experiments were conducted according to the approved protocol (#80006) of the Animal Ethics Committee of Keio University Medical School.

### Methods details

#### Th17-mediated EAD model

The *in vitro* activation procedure of Dsg3H1 T cells was modified from Nishimoto et al.^16^ Lymph node cells from 4–6-week-old Dsg3H1 mice were subjected to magnetic sorting using naive CD4 microbeads (Miltenyi) and LS columns (Miltenyi), following the manufacturer’s protocol. The sorted T cells were cultured in a 24-well tissue culture plate (Corning) coated with anti-CD3 and anti-CD28 mAbs (2 μg/mL each; Biolegend) at a density of 2 × 10^5^/mL in 1 mL of T cell culture media (RPMI medium supplemented with 10% fetal calf serum (FCS), penicillin/streptomycin, non-essential amino acid solution, HEPES solution, sodium pyruvate solution, and 55 μM 2-mercaptoethanol [RPMI and supplements were all procured from Nacalai Tesque, Kyoto, Japan, except 2-ME: Gibco]). To induce pathogenic Th17, mouse IL-6 (20 ng/mL), mouse IL-23 (20 ng/mL), human TGF-β (2 ng), mouse IL-1β (10 ng/mL), anti-mouse IFN-γ (5 μg/mL), and anti-mouse IL-4 (5 μg/mL) were added to the culture. Three days later, cells were harvested from the plate and further expanded in the presence of mouse IL-23 (20 ng/mL) and mouse IL-2 (20 ng/mL) for another 3 days. To induce control Th1, IL-12 (20 ng/mL) and anti-mouse IL-4 (5 μg/mL) were added to the culture. Three days later, cells were harvested from the plate and further expanded in the presence of mouse IL-2 (20 ng/mL) for another 3 days. On the day of transfer, 4–5 million expanded T cells were resuspended in phosphate-buffered saline (PBS) and intravenously injected into 5Gy-irradiated wild-type C57BL/6NCrSlc or non-irradiated B6 CD3e KO mice. In some experiments, donor Dsg3H1 TCR Tg mice were further bred to B6 CD45.1 congenic mice to allow the identification of donor T cells (CD45.1^+^) from recipient cells (CD45.2) *ex vivo*. To evaluate T cell expansion *in vivo*, T cells were labeled with CellTrace Violet dye right before the transfer according to the manufacturer’s instructions. As a humane endpoint, mice with dermatitis were observed for up to 1.5 months and then euthanized.

#### Abatacept and cyanamide treatment

Mice received intraperitoneal injections of abatacept (Bristol-Myers Squibb; 100–200 μg/body) or an equivalent amount of human immunoglobulin (Jackson ImmunoResearch Lab) at the time of transfer and then again on days 2 and 4. In some experiments, mice were administered daily cyanamide (80 mg/kg body weight) dissolved in water via oral gavage. Control mice received water.

#### Preparation of skin infiltrating T cells

For the isolation of skin cells, the pinna of sacrificed mice was mechanically separated into skin and cartilage. The skin was incubated in a 2.5 mg/mL Trypsin/1 mM EDTA solution (Nacalai Tesque) with the epidermal side facing up at 37°C for 1 h and then separated into epidermis and dermis. The epidermal sheet was gently rubbed with the plunger end of a disposable plastic syringe against a 100 mM cell strainer to obtain single cells. The dermis was further digested in RPMI 10% containing FCS, 2 mg/mL collagenase D (Roche), 1.2 mg/mL hyaluronidase (Fujifilm-Wako Pure Chemicals), and 100 μg/mL DNase-I (Roche) at 37°C for 1 h, and single cells were prepared.

#### FACS

Cells from the skin or lymph nodes were stained in FACS buffer (PBS containing 1% BSA and 0.05% sodium azide) with fluorochrome-conjugated antibodies against mouse T cells and a flexible viability dye (Fixable Viability Dye; FVD, Thermo Fisher Scientific). All the antibodies used in this study were obtained from Thermo Fisher Scientific (Tokyo, Japan) or BioLegend (Tokyo, Japan). The stained cells were analyzed using a FACSCanto II analyzer (BD Bioscience) or a CytoFlex S (Beckman), and the data were analyzed using Flowjo software (BD Bioscience). All gating strategy in this study for FACS was presented in Figures S3–S11.

For the detection of intracellular cytokines, cells were cultured at a density of 4 × 10^6^/mL in 1 mL of T cell culture media and stimulated with PMA (50 ng/mL) and ionomycin (1 μg/mL) for 5 h. During the last 2 h of stimulation, brefeldin A (3 μg/mL) and monensin (2 μM) were added to the culture. After the culture, cells were first stained with cell surface antigens, fixed, and permeabilized using a fixation/permeabilization buffer (BD), and then stained with anticytokine antibodies. For the detection of GLUT1, cells were fixed without stimulation, permeabilized in the same way as for cytokines, and then stained with Alexa Fluor 647 anti-GLUT1 antibody.

#### Recovery of donor cells from recipients

For RNA sequencing (Figures 4C and 5A), the restimulation (Figures 4E and 4F) and secondary transfer assay of donor cells (Figures 4G and 4H), spleen and lymph node cells from recipient mice were sorted using CD4 microbeads (Miltenyi) and LS columns (Miltenyi). The sorted CD4^+^ T cells were labeled with a donor T cell marker (CD45.1^+^) and other markers, and then sorted using an FACS ARIA III.

#### T cell restimulation assays

For restimulation by plate-bound antibodies (Figure 3A), Dsg3H1-pTh17 T cells were plated in a 24-well tissue culture plate coated with anti-CD3 and anti-CD28 mAbs (2 μg/mL each) at a density of 1 × 10^6^/mL in 1 mL of T cell culture media. After 2 h, the cells were recovered for RNA extraction.

For restimulation by Dsg3H1 peptide (Figures 4 and S2), 2 × 10^4^ donor T cells were cocultured with 2 × 10^5^ splenocytes from wild-type mice irradiated with 20 Gy in a 96-well flat plate. The co-culture was performed in the presence of 2 μg/mL DsgH1 mimotope peptide (RNKAEFHQSVISQYR) in 0.2 mL of T cell culture media. In Figures 2 and 2 μg/ml anti-CD28 was additionally added to the indicated wells. After three days, cytokine production and cell proliferation were measured using Cytometric Bead Array (BD) and Cell Count Reagent SF, respectively, following the manufacturers’ instructions.

#### Serial transfer model

For the experiment presented in Figures 4G and 4H, spleen and lymph node cells were pooled from 3 to 5 recipients 1 week after transfer. Donor cells were sorted as described above, and then 3 × 10^5^ cells were intravenously transferred to 5Gy-irradiated C57BL/6N mice. The mice were analyzed 1 week later.

#### RNA sequencing

Total RNA was isolated using the RNeasy Plus Micro Kit (Qiagen). Libraries were prepared using the TruSeq stranded mRNA Library kit and sequenced on a NovaSeq 6000 (Illumina) to obtain 150-bp paired-end reads. HISAT2 version 2.1.0 was used to map the RNA-seq data to the mouse genomic DNA sequences (mm10). Read counts, fragments per kilobase of exon per million mapped fragments, and transcripts per million were calculated using featureCounts version 1.6.3. The samples were clustered using the Wald method based on Euclidean distances of the normalized counts, utilizing the stats (Version 3.6.1) and ggplots (Version 3.0.1.1) R packages. Then, DEGs were identified using DESeq2 version 1.30.1.

#### Quantitative reverse transcription-PCR

Total RNA was reverse-transcribed by High-Capacity cDNA Reverse Transcription Kit (Thermo Fisher.) Resulting cDNA was amplified with SsoFast EvaGreen Supermix (Bio-Rad) and CFX Connect Real-time PCR system by according to manufacturers’ protocols. Primers used for PCR are listed in the key resources table.

#### Bioinformatics

Enrichment of GO biological processes was performed using Metascape.^42^ GSEA^43^ was conducted using the GSEA desktop application (ver. 4.2.3). Pathway analysis and molecular characterization information were obtained using Ingenuity Pathway Analysis (IPA; Qiagen).

#### Analysis of human cancer database

Analysis of The Cancer Genome Atlas (TCGA) database for gene correlation was conducted with the assistance of the Timer 2.0 resource.^44^^,^^45^

#### Data deposition

All RNA sequence data have been publicly deposited on NCBI under accession #DRA016062 (run numbers DRR457517-DRR457541).

### Quantification and statistical analysis

All statistical analyses, except for RNA-seq data, were performed using GraphPad PRISM software (ver. 8.4.3). Student’s *T*-test was used for comparing two groups, while multiple comparisons of one-way ANOVA were used for databases involving more than three groups. Cumulative incidence of dermatitis in experiments was analyzed using the Kaplan–Meier method with log rank tests. For gene correlation analysis, partial Spearman’s correlation was determined through TIMER 2.0 analysis. Detailed statistical information for each experiment and the number of replicates can be found in the corresponding figure or figure legends.
