# Supplementary material for: Targeting abatacept-resistant T-helper-17 cells by aldehyde dehydrogenase inhibition
Source: iScience. 2023 Dec 9;27(1):108646. doi: 10.1016/j.isci.2023.108646 (PMC10788227; doi:10.1016/j.isci.2023.108646)

iScience, Volume *27*

Supplemental information

Targeting abatacept-resistant T-helper-17 cells by aldehyde dehydrogenase inhibition

By Yukiko Tokifuji, Hodaka Hayabuchi, Takashi Sasaki, Mariko Hara-Chikuma, Keiji Hirota, Hayato Takahashi, Masayuki Amagai, Akihiko Yoshimura, and Shunsuke Chikuma

Fig S1: *In vitro* Development of pathogenic Th17, related to Fig1A. A. Cytokines produced by cultured Dsg3H1 Th cells. N=3. **B.** Gene expression of normal (n) Th17 and pathogenic (p) Th17. N=4. *T*-test was used for statistical analysis (***: P < 0.0001, ****: P < 0.00001)

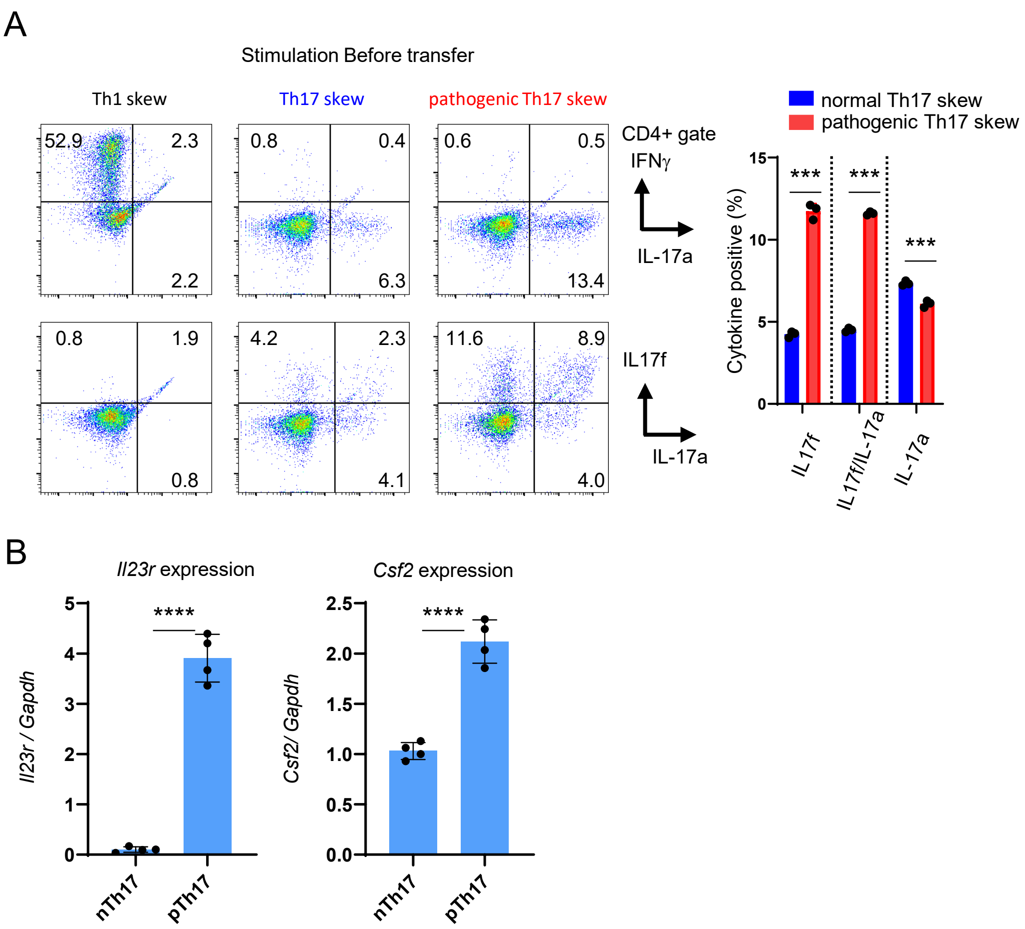

Fig S2: CD28 Functions in pathogenic Th17 *in vitro*, related to Fig3

A. Antigen specific restimulation of Dsg3H1-pTh17. **B and C.** Secondary response of Dsg3H1-pTh17. Cells stimulated by Dsg3H1 mimotope peptide with or without anti-CD28 antibody were compared for secondary proliferation (**B**) and cytokine production (**C**). Data from non-stimulated samples were shown as reference. (Two tailed t-test; N=3).

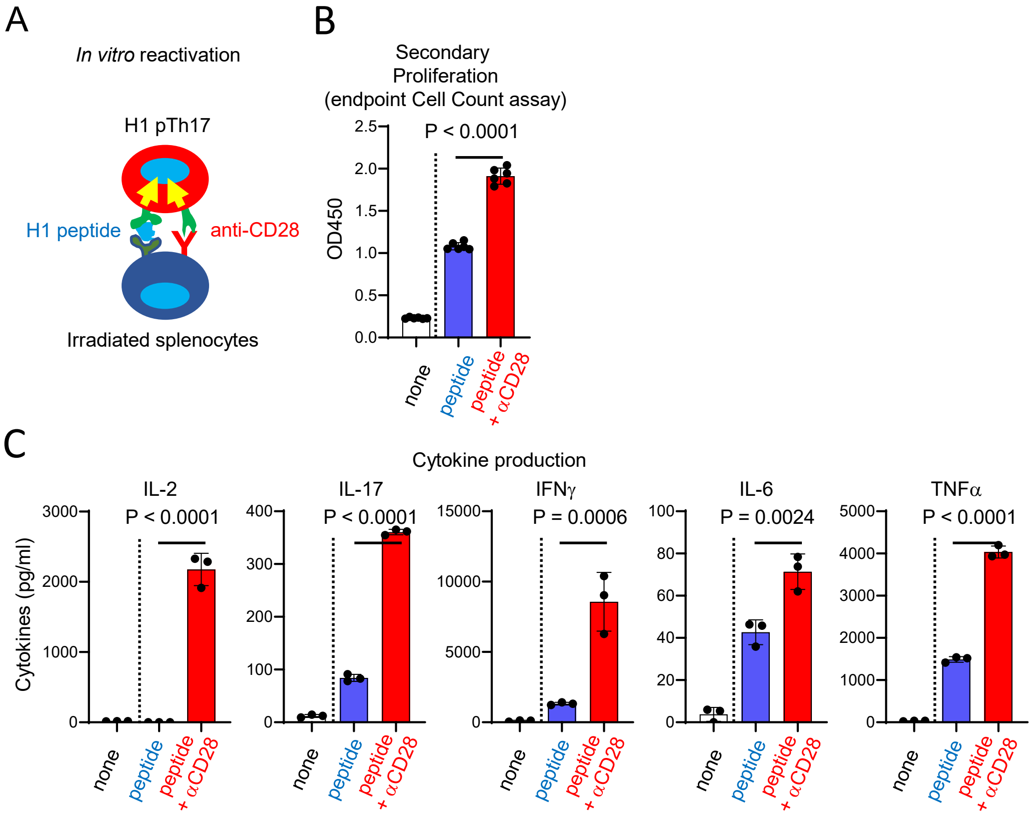

Fig S3: FACS gating strategy related to Fig1A.

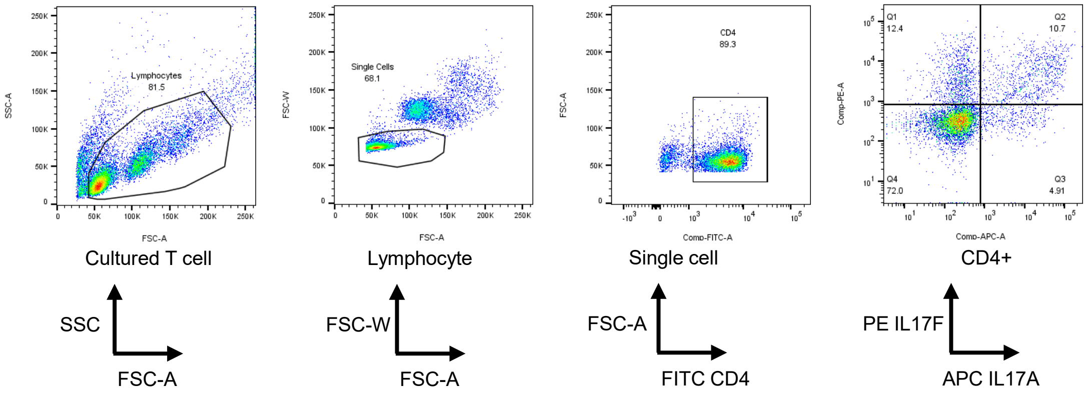

Fig S4. FACS gating strategy related to Fig1E

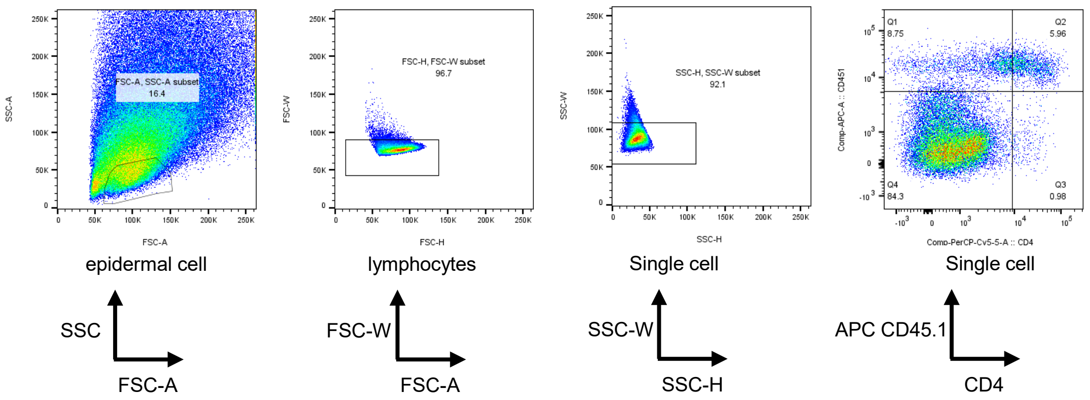

Fig S5. FACS gating strategy related to Fig2C and D.

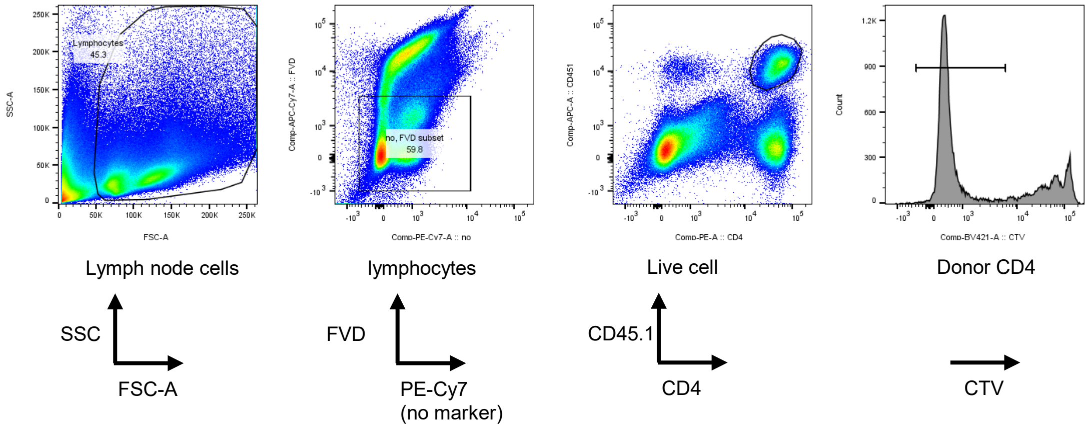

Fig S6. FACS gating strategy related to Fig4A.

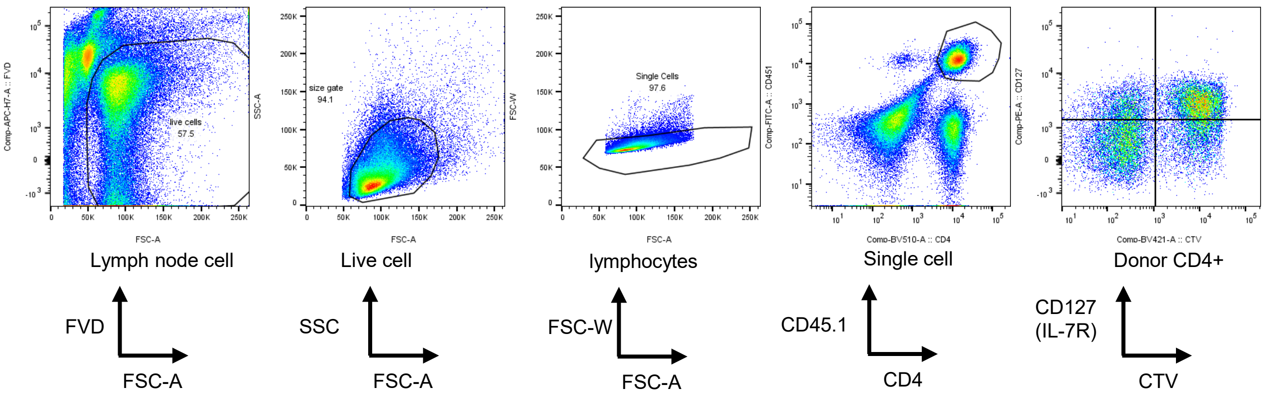

Fig S7. FACS gating strategy related to Fig4C.

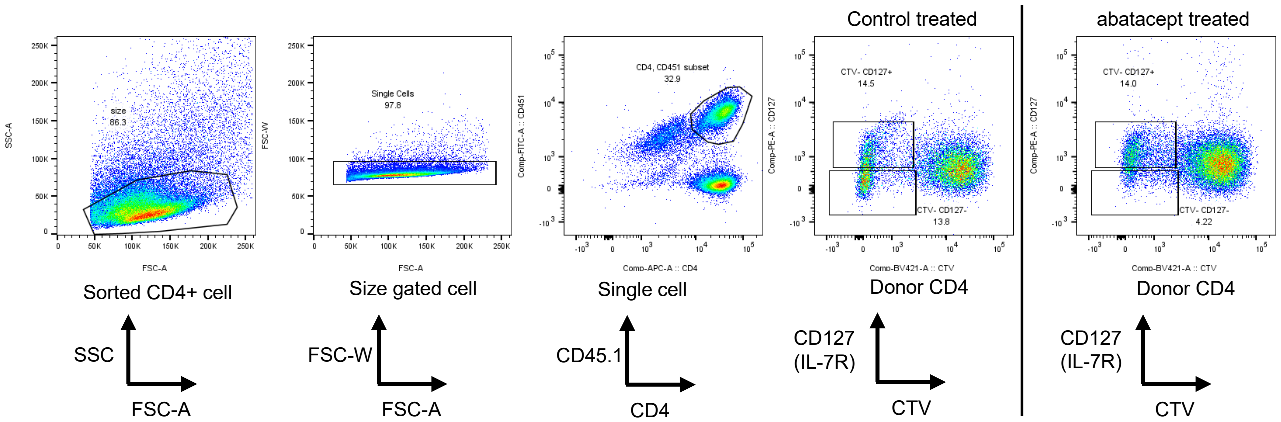

Fig S8. FACS gating strategy related to Fig4H.

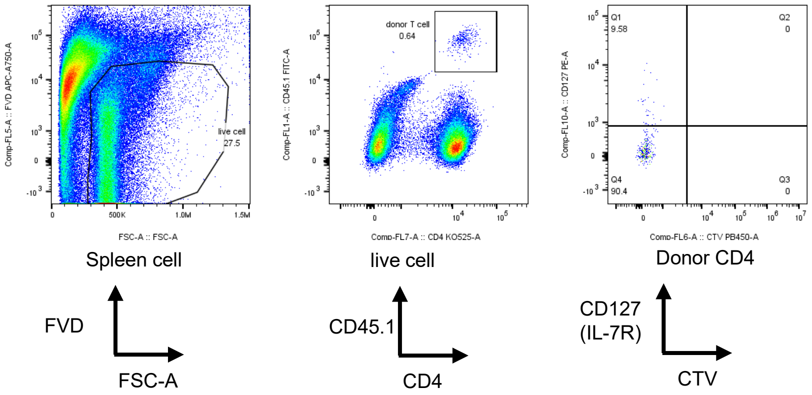

Fig S9. FACS gating strategy related to Fig5A.

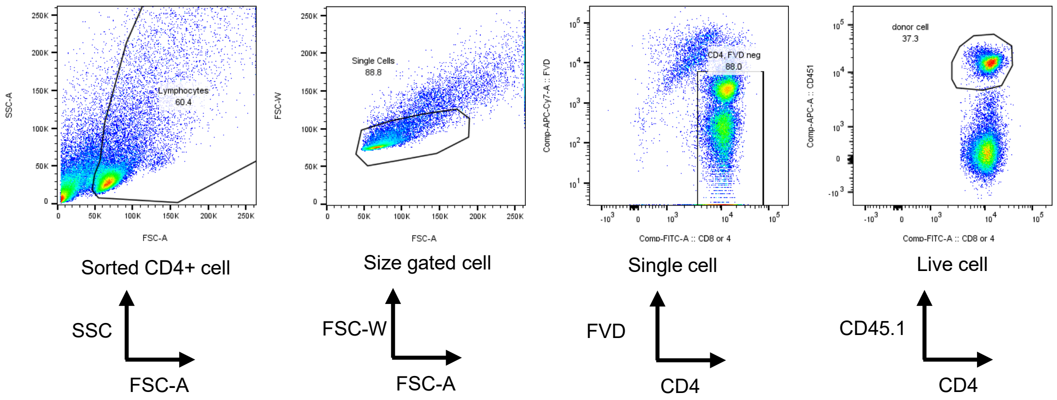

Fig S10. FACS gating strategy related to Fig7C.

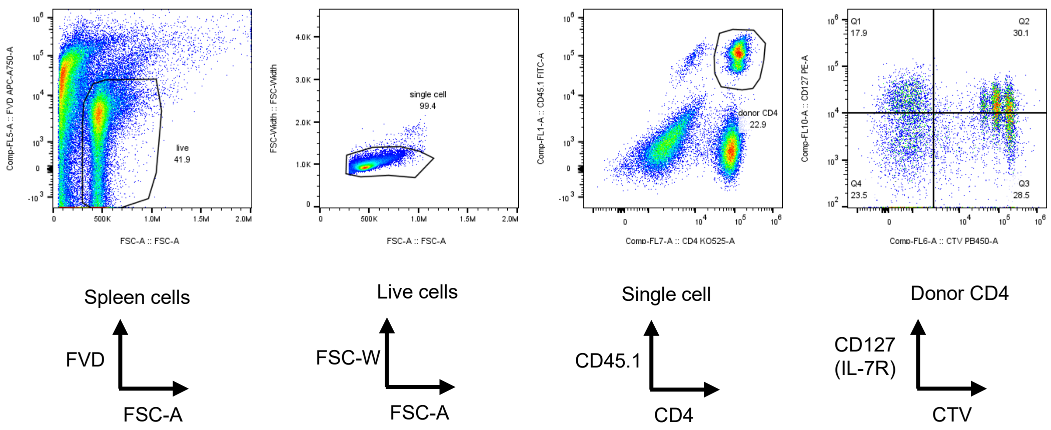

Fig S11. FACS gating strategy related to Fig7D.

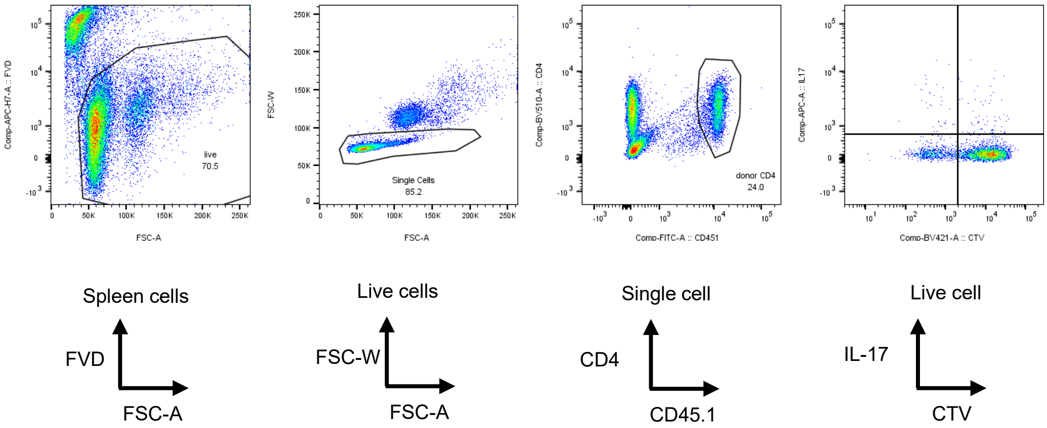

Supplement: Document S1. Figures S1–S11 [file mmc1.pdf]
